# Clinical efficacy of intravenous immunoglobulin therapy in critical ill patients with COVID‐19: a multicenter retrospective cohort study

**DOI:** 10.1002/cti2.1192

**Published:** 2020-10-14

**Authors:** Ziyun Shao, Yongwen Feng, Li Zhong, Qifeng Xie, Ming Lei, Zheying Liu, Conglin Wang, Jingjing Ji, Huiheng Liu, Zhengtao Gu, Zhongwei Hu, Lei Su, Ming Wu, Zhifeng Liu

**Affiliations:** ^1^ Department of Nephrology General Hospital of Central Theater Command of PLA Wuhan 430070 China; ^2^ Department of Critical Care Medicine and Hospital Infection Prevention and Control The Second People’s Hospital of Shenzhen & First Affiliated Hospital of Shenzhen University Health Science Center Shenzhen 518035 China; ^3^ Department of Critical Care Medicine The Third People’s Hospital of Shenzhen Shenzhen 518035 China; ^4^ Department of Critical Care Medicine The First Affiliated Hospital Guizhou University of Chinese Medicine Guiyang 550001 China; ^5^ Department of Critical Care Medicine General Hospital of Southern Theater Command of PLA Guangzhou 510010 China; ^6^ Department of Nephrology Guangzhou Eighth people's hospital Guangzhou Medical University Guangzhou 510060 China; ^7^ Department of Emergency Zhongshan Hospital of Xiamen University Xiamen 361000 China; ^8^ Department of Treatment Center for Traumatic Injuries The Third Affiliated Hospital Academy of Orthopedics Guangdong Province Southern Medical University Guangzhou Guangdong 515630 China; ^9^ Key Laboratory of Hot Zone Trauma Care and Tissue Repair of PLA General Hospital of Southern Theater Command of PLA Guangzhou 510010 China

**Keywords:** SARS‐CoV‐2, COVID‐19, IVIG, clinical efficacy, mortality

## Abstract

**Objective:**

Coronavirus disease 2019 (COVID‐19) outbreak is a major challenge all over the world, without acknowledged treatment. Intravenous immunoglobulin (IVIG) has been recommended to treat critical coronavirus disease 2019 (COVID‐19) patients in a few reviews, but the clinical study evidence on its efficacy in COVID‐19 patients was lacking.

**Methods:**

325 patients with laboratory‐confirmed critical COVID‐19 were enrolled from 4 government‐designated COVID‐19 treatment centres in southern China from December 2019 to March 2020. The primary outcomes were 28‐ and 60‐day mortality, and the secondary outcomes were the total length of in‐hospital and the total duration of the disease. Subgroup analysis was carried out according to clinical classification of COVID‐19, IVIG dosage and timing.

**Results:**

In the enrolled 325 patients, 174 cases used IVIG and 151 cases did not. The 28‐day mortality was improved with IVIG after adjusting confounding in overall cohort (*P = *0.0014), and the in‐hospital and the total duration of disease were longer in the IVIG group (*P* < 0.001). Subgroup analysis showed that only in patients with critical type, IVIG could significantly reduce the 28‐day mortality, decrease the inflammatory response and improve some organ functions (all *P* < 0.05); the application of IVIG in the early stage (admission ≤ 7 days) with a high dose (> 15 g per day) exhibited significant reduction in 60‐day mortality in the critical‐type patients.

**Conclusion:**

Early administration of IVIG with high dose improves the prognosis of critical‐type patients with COVID‐19. This study provides important information on clinical application of IVIG in the treatment of SARS‐CoV‐2 infection, including patient selection and administration dosage and timing.

## Graphical abstract

Early administration of a high dose of intravenous immunoglobulin (IVIG) improves the prognosis of critical‐type patients with COVID‐19. This study provides important information on clinical application of IVIG in the treatment of SARS‐CoV‐2 infection, including patient selection and administration dosage and timing.

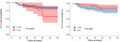

## Introduction

Coronavirus disease 2019 (COVID‐19) is a systemic infectious disease mainly caused by severe acute respiratory syndrome coronavirus 2 (SARS‐CoV‐2), while critical COVID‐19 is a life‐threatening multi‐organ dysfunction syndrome dysregulated resulted from host response to SARS‐CoV‐2 and characterised by refractory hypoxaemia caused by acute respiratory distress syndrome (ARDS).^1^ From December 2019 to April 2020, more than 80,000 people in China got infected with SARS‐CoV‐2, and in which, over 3000 people died. Globally, more than 2.3 million people got infected with SARS‐CoV‐2 and over 150 thousands of people died, including a large number of health workers, which has become the most serious problem faced by all medical staff and researchers.^2^ According to the reports, the general mortality was about 1–15% in all COVID‐19 cases, and the incidence of critical COVID‐19, including both severe and critical types defined by Chinese Recommendations for Diagnosis and Treatment of Novel Coronavirus (SARS‐CoV‐2) infection published by National Health Commission of China (Trial 7th version), is about 10–20%, and its mortality was about 30–60%.^3^, ^4^

It is currently believed that SARS‐CoV‐2 primarily infects the lungs, and subsequently causes systemic inflammation and immune response disorder, and ultimately leads to multiple organ injury and even death.^5^ However, effective therapeutic method is lacking. The available clinical treatment strategies to critical COVID‐19 are mainly antiviral and oxygen therapy, as well as organ and symptomatic support, including mechanical ventilation, even extracorporeal membrane oxygenation (ECMO) of cardiopulmonary support, and continuous renal replacement therapies (CRRT).^3^, ^6^ However, the clinical efficacy of these strategies is still uncertain. Some clinical tests and autopsy results have suggested that the inflammation and immune response caused by the virus infection is the key factor to the progress of disease and the poor prognosis, but the underlying mechanisms remain unclear.^7^, ^8^ The uncontrolled immune response to SARS‐CoV‐2 infection results in a systemic hyperinflammatory response in critically ill COVID‐19 patients.^9^ Hence, immunotherapies based on inflammatory cytokine neutralisation and immunomodulation could reduce inflammation and inflammation‐associated lung damage.^10^ Targeted intravenous immunoglobulin (IVIG) is one of the main treatment measures.^3^, ^6^ However, because of the lack of clinical trials, the efficacy of IVIG has yet to be determined and clinical application is still controversial.

Human Immunoglobulin (pH4) for intravenous injection is a liquid preparation containing human immunoglobulins made from normal human plasma, containing IgG antibody with broad‐spectrum antiviral, bacterial or other pathogens. IVIG can rapidly increase the IgG level in the blood, directly neutralise exogenous antigens and regulate multiple immune functions, including regulating immune media and improving the immune capacity of natural immune cells and lymphocytes. IVIG has been used in the treatment of severe bacterial and viral infection and sepsis.^11^, ^12^ Some studies demonstrated its clinical efficacy, especially in the case of viral infectious diseases,^13^ whereas other studies failed to show its therapeutic efficacy, leading to great controversy on its clinical application in acute respiratory virus infection.^14^ The latest version of China’s therapeutic guidelines of COVID‐19 suggested IVIG as a selective treatment method. However, because of the lack of specific antibodies against SARS‐CoV‐2, the efficacy of IVIG remains to be elucidated. It is noted that the use of IVIG is recommended in the list of selective methods in the COVID‐19 therapeutic guidelines of WHO.^3^, ^6^, ^15^, ^16^

In order to confirm the potential therapeutic efficacy of IVIG to COVID‐19, we retrospectively collected the clinical and outcome data of critical COVID‐19 patients, including both severe type and critical type, from 4 government‐designated treatment centres in three cities (Wuhan, Guangzhou and Shenzhen) in China, and using IVIG as an exposure factor analysed the symptoms and outcomes. Up to date, this is the first clinical multicenter cohort study on IVIG treatment for COVID‐19 with a large number of critically ill patients. This study provides important information on clinical application of the IVIG in the treatment of SARS‐CoV‐2 infection, including patient selection and administration timing and dosage.

## Results

### Demographics and baseline characteristics

Clinical data were collected from 338 confirmed critical COVID‐19 patients. After excluding 13 patients because of missing key information, 325 patients were included in the final analysis (Supplementary figure 1). The detailed demographic and clinical profile data of all critically ill patients with COVID‐19 on baseline are summarised in Table 1. The mean age of patients was 58 years (IQR: 46.0–69.0), and mean body temperature was 37.0°C (IQR: 36.5–37.8). Nearly half of the patients had comorbidities, mainly hypertension (30%), diabetes (12%) and coronary heart disease (10%). Of these, 222 (68%) were severe type and 103 (32%) were critical type. 174 cases used IVIG, and 151 cases did not. Comparisons of baseline characteristics between the two groups showed that the disease was more severe in the IVIG group, presented by older age, higher APACH II (Acute Physiology and Chronic Health Evaluation II) scores and SOFA (Sequential Organ Failure Assessment) scores, higher levels of total bilirubin, direct bilirubin, creatinine, C‐reactive protein, IL‐6 and lactate, but lower platelets and lymphocyte count (all *P* < 0.05), and decreased PaO_2_/FiO_2_ (*P* = 0.011, Table 1).

**Table 1:** Baseline demographics and clinical characteristics and laboratory findings in the IVIG group and non‐IVIG group

	Total (*N* = 325)	IVIG (*N* = 174)	Non‐IVIG (*N* = 151)	*P*‐value
Demographics, clinical characteristics				
Age, years	58.0 (46.0–69.0)	61.0 (50–69.0)	56.0 (38.0–67.0)	0.009
Sex *N* (%)				
Male	189 (58%)	112 (64%)	77 (51%)	0.015
Female	136 (42%)	62 (36%)	74 (49%)	
Comorbidity *N* (%)	155 (48%)	90 (52%)	65 (43%)	0.118
Hypertension	98 (30%)	57 (33%)	41 (27%)	0.272
Coronary heart disease	31 (10%)	24 (14%)	7 (5%)	0.005
Chronic kidney disease	5 (2%)	2 (1%)	3 (2%)	0.541
Diabetes	38 (12%)	21 (12%)	17 (11%)	0.821
Chronic obstructive lung	10 (3%)	5 (3%)	5 (3%)	0.820
Stroke	16 (5%)	10 (6%)	6 (4%)	0.461
Carcinoma	10 (3%)	2 (1%)	8 (5%)	0.049
Other	61 (19%)	40 (23%)	21 (14%)	0.037
Temperature (°C), median (IQR)	37.0 (36.5–37.8)	37.2 (36.6–38.2)	36.8 (36.5–37.5)	< 0.001
Pulse (beats per min), median (IQR)	88 (80.0–97.0)	88.0 (80.0–98.0)	87.5 (79.0–96.0)	0.741
Respiratory rate (breaths per min), median (IQR)	20.0 (20.0–23.0)	20.0 (19.0–23.0)	20.0 (20.0–22.0)	0.197
Systolic blood pressure, median (IQR)	127.0 (117.0–138.0)	129.0 (117.0–138.0)	125.0 (115.0–139.0)	0.656
Diastolic blood pressure, median (IQR)	78.0 (70.0–85.0)	78.0 (70.0–83.0)	79.0 (70.8–86.0)	0.096
APACH II score, median (IQR)	6.0 (4.0–9.0)	7.0 (4.8–9.0)	5.0 (2.0–8.0)	< 0.001
SOFA score, median (IQR)	2.0 (2.0–4.0)	2.0 (2.0–4.0)	2.0 (1.0–3.0)	< 0.001
Clinical classifications *N* (%)				
Severe type	222 (68%)	103 (59%)	119 (79%)	< 0.001
Critical type	103 (32%)	71 (41%)	32 (21%)	
Laboratory findings, median (IQR)				
WBC (1 × 10^9^ L^−1^)	5.8 (4.2–8.3)	5.8 (4.1–8.6)	5.6 (4.3–7.8)	0.907
NEU (1 × 10^9^ L^−1^)	3.9 (2.6–6.5)	4.2 (2.7–7.1)	3.6 (2.4–6.0)	0.089
LYM (1 × 10^9^ L^−1^)	1.0 (0.6–1.4)	0.9 (0.5–1.1)	1.2 (0.8–1.6)	< 0.001
MON (1 × 10^9^ L^−1^)	0.4 (0.3–0.6)	0.5 (0.3–0.6)	0.5 (0.3–0.6)	0.237
PLT (1 × 10^9^ L^−1^)	178.0 (144.0–233.5)	171.0 (135.5–214.3)	191.0 (149.8–246.3)	0.012
HGB (g L^−1^)	129.0 (117.0–141.0)	128.5 (115.8–141.0)	129.0 (117.8–141.5)	0.783
FIB (g L^−1^)	4.1 (3.4–4.8)	4.2 (3.6–4.8)	3.9 (3.0–4.8)	0.020
IL‐6 (pg mL^−1^)	19.1 (7.7–42.8)	23.8 (8.6–52.4)	12.4 (6.2–23.0)	0.005
PCT (ng mL^−1^)	0.1 (0–0.2)	0.1 (0.1–0.2)	0.1 (0–0.1)	0.005
CRP (mg L^−1^)	25.2 (8.7–63.6)	34.1 (13.8–77.0)	15.1 (6.4–38.8)	< 0.001
ALT (U L^−1^)	24.0 (16.1–37.9)	27.3 (18.3–42.0)	22.1 (14.9–36.8)	0.004
TBIL (µmol L^−1^)	11.3 (7.9–15.6)	12.0 (8.3–17.4)	10.3 (7.4–14.3)	0.016
DBIL (µmol L^−1^)	3.7 (2.4–6.1)	4.0 (2.4–6.5)	3.3 (1.9–5.1)	0.007
CREA (µmol L^−1^)	65.0 (52.5–80.9)	67.4 (55.0–85.8)	63.2 (50.0–76.4)	0.032
Lac (mmol L^−1^)	1.6 (1.2–2.2)	1.8 (1.3–2.4)	1.4 (1.0–1.8)	< 0.001
Pa0_2_/FiO_2_	237.9 (164.2–285.0)	215.1 (153.0–277.1)	247.7 (198.4–288.4)	0.083

ALT, alanine aminotransferase; APACHE II, Acute Physiology and Chronic Health Evaluation II; CREA, creatine; CRP, C‐reactive protein; DBIL, direct bilirubin; FIB, fibrinogen; HGB, haemoglobin; IL‐6: interleukin‐6; IQR, interquartile range; Lac, lactic acid; LYM, lymphocyte count; MON, monocytes; NEU, neutrophil; PCT, procalcitonin; PLT, platelet count; SOFA, Sequential Organ Failure Assessment; TBIL, total bilirubin; WBC, white blood cell count.

### Outcomes in all patients

Analysis of primary and secondary outcomes in all patients showed that 42 (13%) died in 28 days and 55 (17%) died in 60 days; death in 60‐day includes 6 (3%) severe‐type patients and 49 (47%) critical‐type patients. In the IVIG group, 22 (13%) died within 28 days and 33 (19%) died within 60 days. In the non‐IVIG group, 20 (13%) died within 28 days and 22 (15%) died within 60 days. There was a significant difference in 28‐day mortality between the IVIG group and the non‐IVIG group after adjusting for age, gender, temperature, systolic blood pressure, comorbidity, PaO_2_/FiO_2_, procalcitonin, C‐reactive protein, white blood cells, neutrophils, lymphocytes, alanine aminotransferase, lactic acid, clinical classifications, SOFA and APACH II (*P* = 0.014) and increases survival time (Supplementary figure 2). Analysis of secondary outcomes in all patients showed that the median time of in‐hospital stay was 20.0 days (IQR: 14.0–28.0), and the total course of the disease was 28.0 days (IQR: 19.0–37.0). Compared between the two groups, both hospital days and total duration of disease were longer in IVIG group, adjusted for confounding factors (both *P* < 0.05, Table 2).

**Table 2:** Effects of IVIG treatment on the primary and secondary outcomes in all patients

	Total (*N* = 325)		IVIG (*N* = 174)	Non‐IVIG (*N* = 151)	*P*‐value	*P*‐value*
Primary outcomes *N* (%)						
28‐day mortality	42 (13%)		22 (13%)	20 (13%)	0.872	0.014
60‐day mortality	55 (17%)		33 (19%)	22 (15%)	0.292	0.469
Secondary outcome, median (IQR)						
In‐hospital days		20.0 (14.0–28.0)	23.5 (16.0–33.0)	16.0 (13.0–22.0)	< 0.001	0.012
Total course of disease^a^		28.0 (19.0–37.0)	31.0 (23.0–39.0)	23.0 (17.0–31.0)	< 0.001	< 0.001

^a^ Total course of disease: time from illness onset to death or discharge, days

* Adjusted for age; gender; temperature; SBP; comorbidity; Pa0_2_/FiO_2_; WBC; NEU; LYM; PCT; CRP; LAC; ALT; clinical classifications; SOFA; APACH II.

### Dosage and timing on outcomes

To further confirm the effects of IVIG dosage on the outcomes of COVID‐19 patients, subgroup with different doses of IVIG (> 15 g per day and ≤ 15 g per day) were compared, and the results showed that high‐dose IVIG (> 15 g per day) significantly reduces 28‐day and 60‐day mortality (*P = *0.044, 0.049, respectively, Table 3), and increases survival time (Supplementary figure 3) as compared with the low‐dose group (≤ 15 g per day).

**Table 3:** Effects of dose and timing of IVIG treatment on the primary and secondary outcomes in all patients

	Total (*N* = 174)	IVIG > 15 g per day (*N* = 74)	IVIG ≤ 15 g per day (*N* = 100)	*P*‐value	
Primary outcomes *N* (%)					
28‐day mortality	22 (13%)	5 (7%)	17 (17%)	0.044	
60‐day mortality	33 (19%)	9 (12%)	24 (24%)	0.049	
Secondary outcome, median (IQR)					
In‐hospital days	23.5 (16.0–33.0)	26.5 (18.0–33.0)	22.0 (16.0–31.0)	0.091	
Total course of disease^a^	31.0 (23.0–39.0)	32.0 (24.0–39.0)	30.0 (22.0–39.0)	0.517	

	Total (*N* = 174)	IVIG > 7 days (*N* = 16)	IVIG ≤ 7 days (*N* = 158)	*P*‐value
Primary outcomes *N* (%)				
28‐day mortality	22 (13%)	3 (19%)	19 (12%)	0.441
60‐day mortality	33 (19%)	7 (44%)	26 (17%)	0.008
Secondary outcome, median (IQR)				
In‐hospital days	23.5 (16.0–33.0)	31.0 (23.0–39.8)	22.0 (16.0–32.0)	0.025
Total course of disease^a^	31.0 (23.0–39.0)	41.5 (31.0–49.0)	30.0 (23.0–38.0)	0.005

^a^ Total course of disease: time from illness onset to death or discharge, days.

To further confirm the effects of IVIG application timing on the outcomes of COVID‐19 patients, subgroups with the time from admission to the beginning of IVIG treatment (> 7 days and ≤ 7 days admission) were compared, and the results showed that early administration of IVIG (≤ 7 days) could significantly reduce 60‐day mortality (*P = *0.008, Table 3), total in‐hospital stay and total course of disease (*P = *0.025 and *P = *0.005, respectively), and significantly increase survival time (Supplementary figure 4).

### Outcomes in subgroups

According to the results of multivariate analysis, deep analysis was carried out in different subgroups. IVIG could significantly decrease the 28‐day mortality of patients in critical type (*P* = 0.009) but had no effects on the 60‐day mortality and the length of in‐hospital stay (both *P *> 0.05, Table 4). However, in the severe‐type patients, there was no difference in mortality between the IVIG group and the non‐IVIG group (*P *> 0.05), and the length of in‐hospital stay in the IVIG group did not change (Table 4). Moreover, there was no difference in the 60‐day survival rate between the two groups (Supplementary figure 5).

**Table 4:** Effects of IVIG treatment on the primary and secondary outcome analysis in subgroup of critical and severe types

	Critical type		*P*‐value	Severe type		*P*‐value
	IVIG (*N* = 71)	Non‐IVIG (*N* = 32)		IVIG (*N* = 103)	Non‐IVIG (*N* = 119)	
Primary outcome *N* (%)						
28‐day mortality	19 (27%)	17 (53%)	0.009	3 (3%)	3 (3%)	0.858
60‐day mortality	30 (42%)	19 (60%)	0.107	3 (3%)	3 (3%)	0.858
Secondary outcome						
In‐hospital days	27 (15.0–35.0)	17 (11.5–22.0)	0.005	22 (18.0–30.0)	15 (13.0–22.0)	< 0.001
Total course of disease^a^	33 (21.0–43.0)	29 (23.3–36.0)	0.272	30 (23.0–37.0)	20 (16.0–29.0)	< 0.001
	**IVIG > 15 g per day (*N* = 40)**	**IVIG ≤ 15 g per day (*N* = 31)**		IVIG > 15 g per day (*N* = 34)	**IVIG ≤ 15 g per day (*N* = 69)**	
Primary outcomes *N* (%)						
28‐day mortality	5 (13%)	14 (45%)	0.002	0	3 (4%)	0.217
60‐day mortality	9 (23%)	21 (68%)	< 0.001	0	3 (4%)	0.217
Secondary outcome, median (IQR)						
In‐hospital days	28 (18.3–36.0)	16 (7.0–33.0)	0.011	22 (18.0–30.0)	23 (18.0–31.0)	0.83
Total course of disease^a^	35.5 (27.3–42.5)	26 (14.0–47.0)	0.034	27.5 (23.0–35.0)	34 (25.0–39.0)	0.091

^a^ Total course of disease: time from illness onset to death or discharge, days.

Further comparison between the subgroups with different COVID‐19 types showed that high‐dose IVIG of more than 15 g per day could significantly reduce the 28‐day and 60‐day mortality of the critical‐type patients (*P* = 0.002, *P* < 0.001, respectively, Table 4). In contrast, in the severe‐type patients, neither high dose nor low dose of IVIG demonstrated any effects (Table 4).

To further confirm the efficacy of IVIG on primary outcomes, the Cox proportional hazards model was used with a crude model adjusted for none, Adjust I model adjusted for age, gender, PaO_2_/FiO_2_ and comorbidity, and Adjust II model adjusted for age, gender, PaO_2_/FiO_2_, comorbidity, interleukin‐6, procalcitonin, platelets, lymphocytes, lactic acid, SOFA and APACH II. The results showed that IVIG significantly reduces 28‐day mortality only in critical‐type patients [HR; 0.4 (0.2, 1.0), *P = *0.046, Table 5], and there was no significant difference in 60‐day mortality, regardless of whether the patient was critical or severe (all *P *> 0.05, Table 5).

**Table 5:** Efficacy of IVIG on primary outcomes in subgroups of critical and severe types

	Total HR (95% CI)	*P*‐value	Critical type HR (95% CI)	*P*‐value	Severe type HR (95% CI)	*P*‐value
28‐day mortality						
Non‐adjusted	0.5 (0.3, 0.9)	0.028	0.4 (0.2, 0.8)	0.014	1.1 (0.2, 5.6)	0.889
Adjust I	0.6 (0.3, 1.2)	0.143	0.4 (0.2, 1.0)	0.046	3.7 (0.5, 27.5)	0.194
Adjust II	0.4 (0.1, 2.7)	0.327	0.0 (0.0, 0.4)	0.009	4.1 (0.0, Inf)	1.000
60‐day mortality						
Non‐adjusted	0.6 (0.4, 1.1)	0.100	0.6 (0.3, 1.0)	0.064	1.1 (0.2, 5.6)	0.889
Adjust I	0.7 (0.4, 1.3)	0.225	0.6 (0.3, 1.1)	0.086	3.7 (0.5, 27.5)	0.194
Adjust II	0.5 (0.1, 3.1)	0.473	0.1 (0.0, 0.8)	0.028	4.1 (0.0, Inf)	1.000

Non‐adjusted model adjusted for none.

Adjust I model adjusted for age; gender; PaO2/FiO2; comorbidity.

Adjust II model adjusted for age; gender; PaO2/FiO2; comorbidity; IL‐6; PCT; LYM; PLT; LAC; SOFA; APACH II.

## Discussion

The pandemic outbreak of COVID‐19 is rapidly spreading all over the world, resulting in over one million global deaths because of no well‐established treatment. To our knowledge, this multicenter retrospective cohort study is the first clinical evaluation, with a large number of cases, on the efficiency of IVIG treatment to critical COVID‐19 patients. The basic condition of patients in the IVIG group was more serious. The results showed that, for the critical COVID‐19 patients, IVIG has no effect on the 28‐day and 60‐day mortality. Notably, multivariable regression showed that both classification of COVID‐19 and using IVIG were the factors that were related to the hazards ratios of death. Subgroup analysis showed that only in the critical‐type patients, IVIG could significantly decrease the inflammatory response, improve some organ functions, reduce the 28‐day mortality rate and prolong the survival time. Furthermore, the study showed that the early use of IVIG (admission ≤ 7 days) with high dose (> 15 g per day) exhibits a more significantly curative effect. Noteworthy, the results indicated that the early and high dose of IVIG therapy seems only effective in the critical‐type patients, showing an improved prognosis. These findings provide important information on clinical application of the IVIG in the treatment of SARS‐CoV‐2 infection, including patient selection and administration timing and dosage.

Excessive inflammation is one of the major causes of pathology, and severe cytokine storm has been found to be related to increased death rates in critical COVID‐19 patients.^17^ Targeted anti‐inflammatory responses, such as antimalarials, anti‐IL‐6, anti‐IL‐1 and IVIG, are being evaluated to reduce inflammation‐induced damage.^9^, ^18^ Regarding the clinical application of immunoglobulin in COVID‐19 patients, including the efficacy and use (timing and dosage) is still controversial. Pharmacological studies have suggested that a high dose of IVIG pulse therapy leads to the formation of an immunocomplex with pathogen antigen, which can be further cleared in the circulation.^13^ Immunoglobulin has been used in the treatment of viral infectious diseases, such as viral pneumonia, and autoimmune diseases.^14^ Animal experiments have shown that IVIG could decrease the pro‐inflammatory cytokines’ concentrations in septic mice.^19^ Data from animal inflammation models and human cells showed that IVIG could enhance the regulatory T‐cell proliferation and suppress pro‐inflammatory Th17 cells,^20^, ^21^ with decreased pro‐inflammatory cytokines (such as IL‐17A and IL‐6) and increased anti‐inflammatory cytokines (such as IL‐10).^22^, ^23^ We also found that, after IVIG treatment, the IL‐6 concentration in plasma was decreased in the COVID‐19 patients (data were not shown), suggesting that the benefits by IVIG might be associated with reduced inflammation. Since patients received multiple treatments, this present observational study is not conclusive, and randomised controlled trials are required in the future. In patients with severe COVID‐19, but not in patients with mild disease, lymphopenia is a common feature, with drastically reduced numbers of CD4^+^ T cells, CD8^+^ T cells, B cells and natural killer (NK) cells.^5^, ^24^, ^25^ Immunoglobulin showed antiviral and anti‐inflammatory effects through increasing certain cytokine secretion, such as IL‐2, to promote T‐cell and B‐cell proliferation and differentiation.^13^, ^26^ Therefore, immunoglobulin is thought to be beneficial in the treatment of COVID‐19.^16^ Previous studies in the treatment of SARS and MERS suggested a beneficial role for administration of high‐dose immunoglobulin.^27^, ^28^ In general, IVIG showed multiple effects in immune regulation, not only suppressing the pro‐inflammatory cells activation, but also indirectly enhancing T‐cell and B‐cell proliferation by cytokines.

Since immunoglobulin is not the specific antibody to any virus and the clinical evidence for its efficacy is limited, some researchers hold the opposing view about the usage of immunoglobulin in acute virus infection.^29^, ^30^ In COVID‐19 treatment guidance by China and WHO, the recommendation on immunoglobulin usage is different. The data from the current study showed that IVIG did not improve the all‐cause mortality in enrolled patients. However, subgroup analysis showed that IVIG could only improve the prognosis in the critical‐type patients, suggesting that IVIG showed more benefit for those patients with severe conditions, similar to results from severe influenza patients and MERS patients.^31^ Our results showed that IVIG treatment could decrease the 28‐day fatality of the critical‐type COVID‐19 patients. However, there was no difference in the 60‐day fatality between the IVIG and non‐IVIG groups, and the mechanism is unknown. One of the potential explanations might be that the patients who died within 28 days were older, with higher APACHE II and SOFA scores, higher critical‐type proportion and lower oxygenation indices. These results suggest that those patients who died within 28 days after infection suffered a more severe inflammatory condition. Since IVIG targets the immune responses, these patients may benefit more from IVIG treatment. For those deaths after 28 days, immune dysregulation may not the main cause of death. Long duration of COVID‐19 infection is associated with more complications, and so it is which is difficult to judge the therapeutic effect of IVIG. In addition, among the 55 deaths in our data, only 13 cases died between 28 and 60 days. Therefore, the limited number of cases means that an effective statistical conclusion cannot be drawn.

The recommended dose of immunoglobulin is 0.5 g/kg per day. However, in the present study, the doses used differ among the different centres and physicians, ranging from 0.1 to 0.5 g/kg per day. The treatment period ranged from 5 to 15 days. By subgroup analysis, we found that only high dose over 15 g per day (equivalent to 0.2–0.3 g/kg per day) shows the curative effect, which is consistent with the usage of immunoglobulin in treating sepsis is effective only when administered in high dose.^32^ High‐dose IVIG reduces the activation of innate and adaptive effector immune cells. IVIG, although containing antibodies to various foreign antigens, benefits most likely in critical COVID‐19 cases as a result of its action on inflammation.

The current study suggests the importance of the early use of immunoglobulin in the COVID‐19 patients. Immunoglobulin affects both innate and adapted immune systems, and directly binds to pathogen antigen, which usually appears in the circulation in an early stage following virus infection.^33^, ^34^, ^35^ Based on the current understanding of the COVID‐19 pathogenesis, in late stages, excessive inflammatory response is developed, and organ dysfunction occurs, so the efficacy of administration of immunoglobulin would be largely limited.^7^, ^8^ Our data showed that immunoglobulin employed within 7 days after hospital admission could improve the prognosis. Also, we found that patients who were enrolled with IVIG group were more severe ill, as evidenced by higher APACHE II and SOFA scores, higher levels of IL‐6 and lactate, and decreased lymphocyte count and oxygenation index in this multicenter retrospective study.

Compared to SARS and MERS, COVID‐19 demonstrates several exceptionalities, such as prolonged course, potential asymptomatic hypoxia, and severe lung injury.^3^, ^4^, ^36^ These clinical features urgently call for exploratory treatment attempts. IVIG is one of such attempts. To exclude the influence of the bias on the study, we performed the regression analysis on the potential factors. Univariate survival analysis showed that APACHE II and SOFA scores were the risk factors which were related to the outcome. In further analysis, we found APACHE II and SOFA scores were relatively low in most enrolled patients. This is consistent with the characteristics of this disease, that is only in the patients with severe lung injury, but few injuries to other organs. Cox regression analysis confirmed that critical‐type COVID‐19 patients showed poor prognosis and IVIG improved their survival rate. Although IVIG does not show a therapeutic effect on the whole cohort, it can be beneficial to the critical‐type patients. In addition, Cox regression analysis also showed that lymphopenia was the risk of poor prognosis. This observation is consistent with the previous study reported that 35–83% of COVID‐19 patients showed decreased lymphocyte count, and persistent lymphopenia was related to the poor outcome.^36^ However, subgroup analysis based on the lymphocyte counts did not show an improved outcome related to the IVIG intervention. The explanation for this is uncertain. Future study on the role of IVIG in regulating lymphocyte number and function is needed.

There are some limitations in present study. First, the cases from these 4 clinical centres may still lack sufficient representation. Second, the dose and timing of IVIG administration in each centre may not be exactly consistent. Third, limited by the clinical workload and situation, the evaluation of immunoglobulin effect is mainly based on the clinical manifestations, rather than direct cellular and molecular assessment, including viral load and lymphocyte activation. With the progression in recognition of COVID‐19, large cases with randomised control studies and more developed evaluation systems are needed to confirm the efficiency of IVIG on COVID‐19 treatment.

In conclusion, the present study is the first clinical research evaluating the efficiency of IVIG treatment to critical COVID‐19 patients. The data demonstrate that early application of high‐dose IVIG can improve the prognosis of COVID‐19 patients with critical type. This study provides important information on clinical application of IVIG in the treatment of SARS‐CoV‐2 infection, including patient selection and administration of timing and dosage.

## Methods

### Study design and participants

This multicenter retrospective cohort study was performed in 4 government‐designated treatment centres for COVID‐19 patients in 3 cities in China, including Wuhan, Guangzhou and Shenzhen. The data collection period was from December 2019 to March 2020. The study was approved by the Research Ethics Commission of General Hospital of Southern Theater Command of PLA (HE‐2020‐08), and the requirement for informed consent was waived by the Ethics Commission.

Inclusion criteria are as follows: (1) adults ≥ 18 years old; (2) laboratory (RT‐PCR)‐confirmed SARS‐CoV‐2 infection in throat swab and/or sputum and/or lower respiratory tract samples; or conformed plasma positive of specific antibody (IgM or/and IgG) against SARS‐CoV‐2; (3) in‐hospital treatment ≥ 72 hours; and (4) meet any one of the following a–c criteria for severe type or d–f criteria for critical type: (a) respiratory rate ≥ 30 min^−1^; or (b) rest SPO_2_ ≤ 90%; or (c) PaO_2_/FiO_2_ ≤ 300 mmHg; or (d) respiratory failure and needing mechanical ventilation; or (e) shock occurs; or (f) multiple organ failure and needing ICU monitoring.

Exclusion criteria are as follows: (1) existence of other evidence that can explain pneumonia, including but not limited to influenza A virus, influenza B virus, bacterial pneumonia, fungal pneumonia and noninfectious causes and (2) women who are pregnant or breastfeeding.

### Procedures

We designed the data collection form, which includes the demographic, clinical, treatment, laboratory data and prognosis were extracted from electronic medical records. Detailed clinical data before and after prescription IVIG, and the data at the corresponding time of the same period in non‐IVIG group were collected, respectively. Whether and when to use IVIG, dosage and course were decided by the doctors in charge. Comparison was conducted according to whether IVIG was used or not. Primary endpoints were 28 days and 60 days in‐hospital mortality, and total in‐hospital days and the total duration of the disease were the secondary endpoints. Analysis of the outcome and the survival curves were carried out according to clinical classification of COVID‐19, IVIG dosage and timing. IVIG represents the human immunoglobulin (pH4) for intravenous injection (produced by Shanghai RAAS Blood Products Co., Ltd), which is a liquid preparation containing human immunoglobulins made from normal human plasma, containing IgG antibody with broad‐spectrum antiviral, bacterial or other pathogens. The doses used differed among the different centres and physicians, ranging from 0.1 to 0.5 g/kg per day for infusion. The treatment period ranged from 5 to 15 days.

### Definitions

'Critical COVID‐19' in this article is defined to be a combined term of 'severe type' and 'critical type' of COVID‐19, classified following Chinese Recommendations for Diagnosis and Treatment of Novel Coronavirus (SARS‐CoV‐2) infection (Trial 7th version) published by National Health Commission of China. IVIG represents the human immunoglobulin for intravenous injection, which is a liquid preparation containing human immunoglobulins made from normal human plasma, containing IgG antibody with broad‐spectrum antiviral, bacterial or other pathogens. IVIG rapidly increases the level of IgG in the blood of the recipient after intravenous infusion and enhances the anti‐infection ability and immune regulation function of the body.

### Statistical analysis

The categorical data were summarised as numbers and percentages, and intergroup comparisons were performed using the Mann–Whitney *U‐*test, chi‐square tests or Fisher’s exact test. Continuous variables were expressed as the arithmetic mean and standard deviation (SD) or as the median and interquartile range, depending on whether they showed a Gaussian distribution. Continuous data with Gaussian distribution were compared with the Student’s *t*‐test or one‐way ANOVA and those with a non‐Gaussian distribution with the Wilcoxon rank‐sum test. To determine the primary and secondary outcomes in patients after adjusting for confounders, the Cox proportional hazards model was used with a fully adjusted model: HR (hazards ratio) and 95% confidence interval levels (95% CI). For analysis of the 28‐day and 60‐day mortality, Kaplan–Meier survival curves and the log‐rank test were used. Statistical analysis was performed using the SPSS Windows version 11.0 (SPSS Inc, Chicago, IL), and Empower (R) (http://www.empowerstats.com, X&Y solutions, Inc., Boston, MA) and R (http://www.R‐project.org) software, and *P*‐values (two‐tailed) below 0.05 were considered to be statistically significant.

### Ethics approval and consent to participate

The study was approved by the Research Ethics Commission of General Hospital of Southern Theater Command of PLA (HE‐2020‐08), and the requirement for informed consent was waived by the Ethics Commission.

## Conflict of Interest

The authors declare no conflict of interest.

## AUTHOR CONTRIBUTIONS

LZ, WM, SL, FY and HZ: Study concept and design. SZ, WM, XQ, LH, GZ, LZ, LZY ZL and WC: Data collection. WM, LZ, LZY ZL and WC: Statistical analysis. LZ, WM, JJ and ZJ: Preparation of draft of the manuscript. All authors: Data access; data integration; accuracy of data analysis.

## Funding

This work was supported by grants from the PLA Logistics Research Project of China [18CXZ030, CWH17L020 and 17CXZ008], Sanming Project of Medicine in Shenzhen (SZSM20162011) and Clinical Research Project of Shenzhen Municipal Health Commission (SZLY2017007).

## Consent for publication

All authors reviewed the manuscript and approved the publication.

## Data Availability

The data sets used and/or analysed during the current study are available from the corresponding author on reasonable request.
