# Supplementary material for: Clinical efficacy of intravenous immunoglobulin therapy in critical ill patients with COVID‐19: a multicenter retrospective cohort study
Source: Clin Transl Immunology. 2020 Oct 14;9(10):e1192. doi: 10.1002/cti2.1192 (PMC7557105; doi:10.1002/cti2.1192)
Supplement: Supplementary file 1 [file CTI2-9-e1192-s001.docx]

**Supplementary figure 1.** Flow diagram of study subjects**.**

338 patients

13 patients excluded by missing data

325 patients included

IVIG group

N=174

Non-IVIG group

N=151

**Supplementary figure 2.** Effects of IVIG treatment on 28-day mortality and 60-day mortality in all patients.


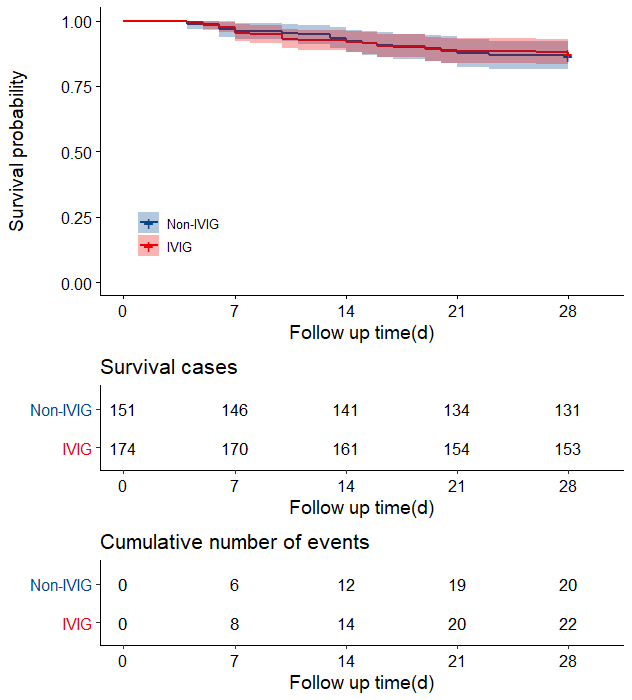


*P**=0.014


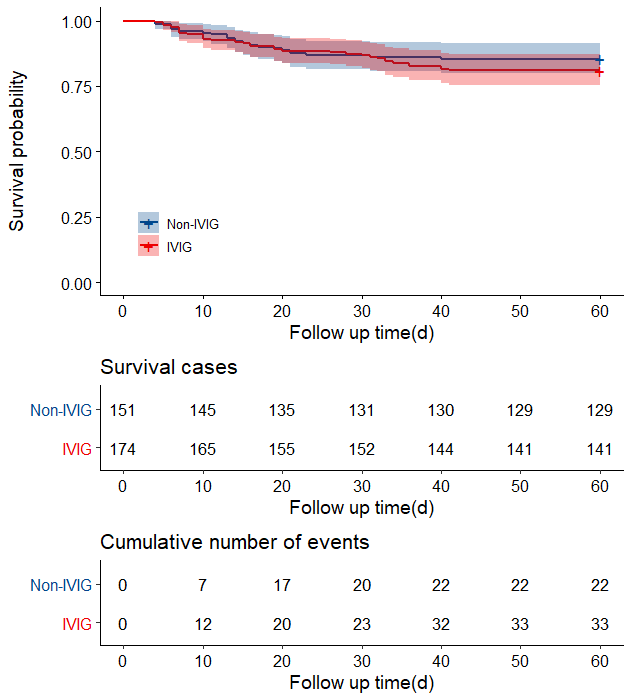


*P**=0.469

**Supplementary figure 3.** Dose of IVIG treatment on 28-day mortality and 60-day mortality in all patients.


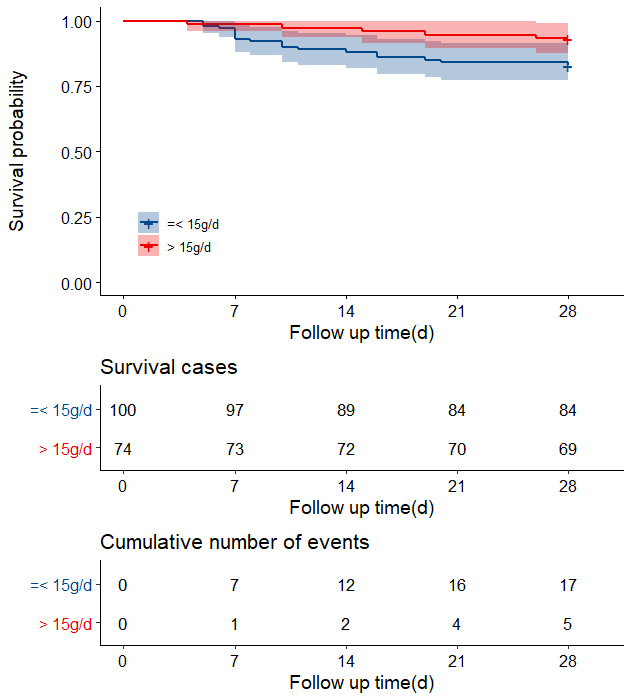

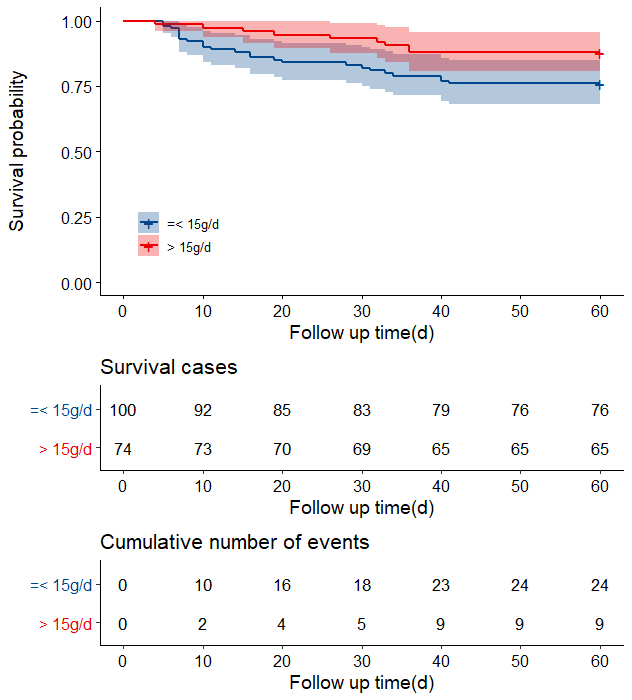


*P* =0.044

*P* =0.049

**Supplementary figure 4.** Timing of IVIG treatment on 28-day mortality and 60-day mortality in all patients.


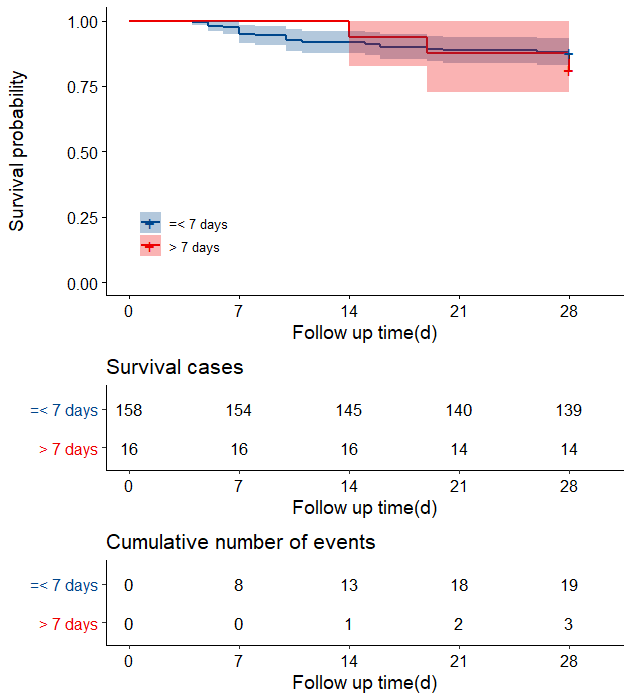

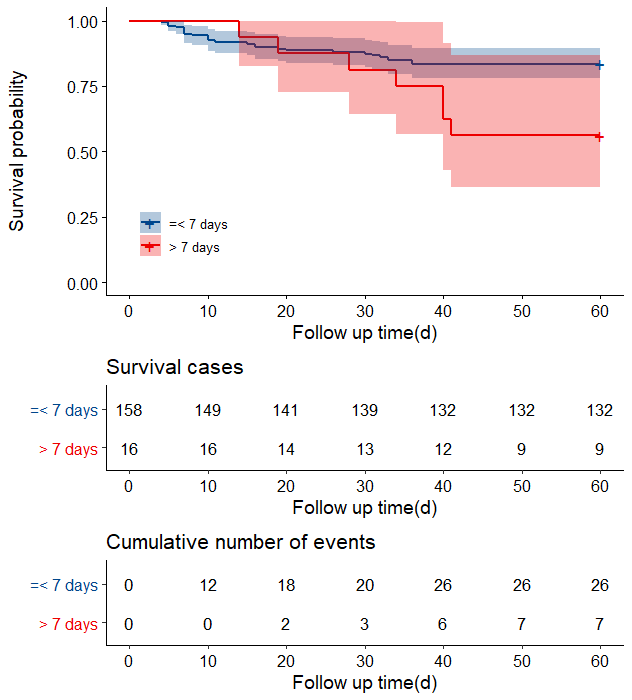


*P* =0.441

*P* =0.008

**Supplementary figure 5.** Effects of IVIG treatment on 28-day mortality in patients with critical type and severe type.


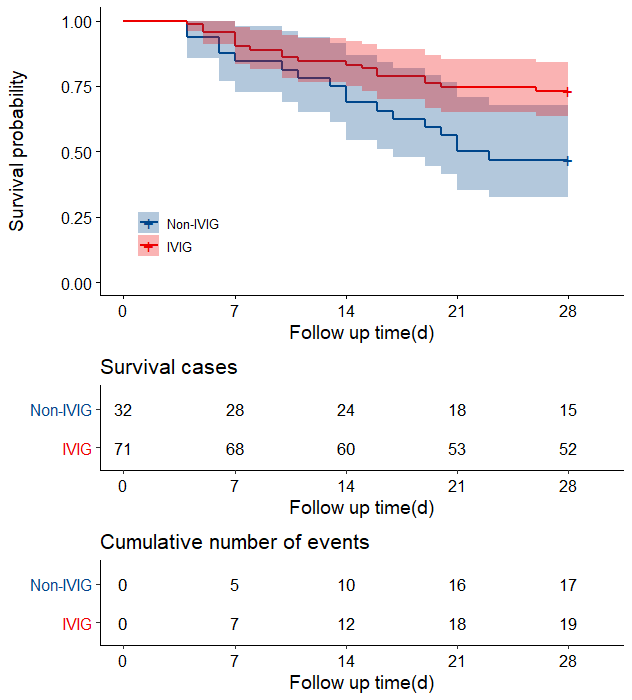

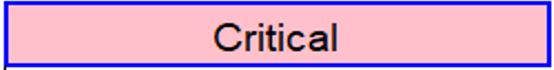

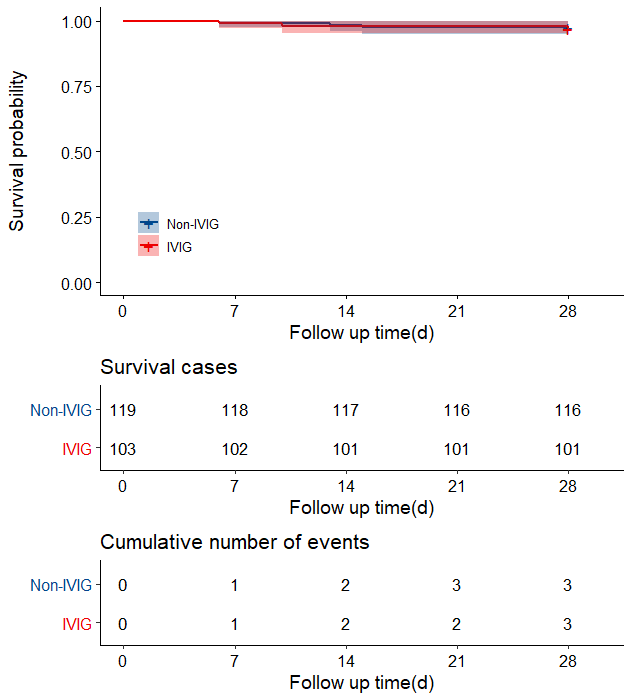

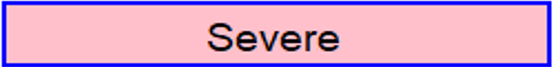


*P* =0.009

*P* =0.858
